# “There Is No Link Between Resource Allocation and Use of Local Data”: A Qualitative Study of District-Based Health Decision-Making in West Bengal, India

**DOI:** 10.3390/ijerph17218283

**Published:** 2020-11-09

**Authors:** Sanghita Bhattacharyya, Anns Issac, Bhushan Girase, Mayukhmala Guha, Joanna Schellenberg, Bilal Iqbal Avan

**Affiliations:** 1 Public Health Foundation of India, Gurgaon, Haryana 122002, India; Sanghita@phfi.org (S.B.); annsissac@gmail.com (A.I.); bhushangirase@gmail.com (B.G.); mayukhmala21@gmail.com (M.G.); 2 London School of Hygiene and Tropical Medicine, Keppel Street, London WC1E 7HT, UK; Joanna.Schellenberg@lshtm.ac.uk

**Keywords:** decision-making, district health system, health administration, health management information system, maternal and child health

## Abstract

Background: Effective coordination among multiple departments, including data-sharing, is needed for sound decision-making for health services. India has a district planning process involving departments for local resource-allocation based on shared data. This study assesses the decision-making process at the district level, with a focus on the extent of local data-use for resource allocation for maternal and child health. Methods: Direct observations of key decision-making meetings and qualitative interviews with key informants were conducted in two districts in the State of West Bengal, India. Content analysis of the data maintained within the district health system was done to understand the types of data available and sharing mechanisms. This information was triangulated thematically based on WHO health system blocks. Results: There was no structured decision-making process and only limited inter-departmental data-sharing. Data on all 21 issues discussed in the district decision-making meetings observed were available within the information systems. Yet indicators for only nine issues—such as institutional delivery and immunisation services were discussed. Discussions about infrastructure and supplies were not supported by data, and planning targets were not linked to health outcomes. Conclusion: Existing local data is highly under-used for decision-making at the district level. There is strong potential for better interaction between departments and better use of data for priority-setting, planning and follow-up.

## 1. Introduction

Decision-making in health systems involves stakeholders reaching consensus on a particular course of action from two or more possible options to address health service challenges. At the district level, local decision-making depends on autonomy over resource allocation and in the planning process [1]. Devolution of administrative powers for the department of health services can result in giving district-level decision-makers a higher degree of autonomy over finances, service distribution, human resources and governance [2]. In contrast, in countries with a centralised decision-making process, local needs might not adequately be reflected in resource allocation [3]. One of the key aspects of a decision-making process is to have a well-structured strategy for problem recognition and building consensus among all stakeholders towards a solution [4,5]. A coordinated process of decision-making may not happen if there is a power imbalance among the stakeholders and a lack of clarity around roles within the group [6,7].

Theoretically, a decentralised health system creates opportunities for managers to make decisions that are innovative and meet local needs, which can result in the improvement of service delivery [8]. To make independent and local decision-making opportunities work, there is a need for synergy, expanding capacities and strengthening accountability mechanisms [9]. There is variation in the use of decision-making opportunities across district-level officials: some make greater use of decision space than others, and those who do so also tend to have more capacity [2]. Local decision-making empowers district-level staff to have a direct impact on key indicators of health systems performance [10]. Within the government health system in low and middle-income countries (LMIC), there are multiple departments, in addition to health departments, that deliver services related to public health. Effective coordination between these departments, including sharing information, is needed to support comprehensive local decision-making [11,12]. Moreover, the success of the decision-making space is contingent upon contextual factors, such as cooperation among members, leadership capabilities, involvement in the day-to-day functioning of health units, sense of ownership of the health units, interests of decision-makers, and involvement of the community [13]. There is a need for staff to take ownership of the programmes and have a clear understanding of their roles and responsibilities [10].

Ideally, the planning and problem-solving process in a district should not only depend on well-defined coordination among the decision-makers, but also on the use of data from the health management information system (HMIS). Studies in LMICs have identified several mechanisms in which data are used locally in structured processes to make decisions. Examples of these mechanisms include routine data quality audits, identifying gaps in data, timely feedback on health system performance through summary data dashboards, and routine data review meetings [5,14]. There is limited systematic evidence related to maternal and child health (MCH) services about how data were used for decision-making at the district management level. Bhattacharyya and Murray highlighted the use of local health data at the community level for planning and monitoring MCH [15]. Yet often data are not used optimally for routine planning, monitoring and evaluation, due to inadequate sharing of complete, accurate and timely data; duplicate and parallel reporting channels, and insufficient capacity to analyse and use data for decision-making [14,16,17,18,19,20]. 

In India since 2005, through the Health Sector Reform Programme, the National Health Mission has sought to decentralise planning and increase community involvement, particularly in planning and decision-making at district level [21]. The National Health Mission further aims to integrate district health plans with those of sectors, which provide health-related services, such as the Departments of Women and Child Development, Rural Development and Education [22]. Additionally, all these sectors generate health data, which are systematically collected from the community to the district level [23]. Yet few studies have examined the perceptions and experiences of local decision-makers [13].

### Context: Health System Connectivity at the District Level

This study was undertaken in West Bengal, the fourth most populous of India’s 29 states, with a population of more than 90 million. West Bengal has an infant mortality rate of 28 per thousand live births, whereas the national average is 39 [24], and the maternal mortality rate is 117 per 100,000 live births, compared with 178 for the whole of India [25].

The Health and Family Welfare Department of the State Government is responsible for the health-care system and is divided into three tiers. These are the primary health care network, comprising community and primary health centers; the secondary care system, comprising district and sub-divisional hospitals, and the tertiary hospitals, providing speciality and super-speciality care. The Chief Medical Officer of Health (CMOH) heads the health administration in a district and is responsible for the effective implementation of the various medical, health and family welfare programmes, including planning, supervision and coordination [26].

The CMOH also supervises the functioning of the National Health Mission’s District Programme Management Unit, which assists in preparing plans, an annual budget and in maintaining accounts. CMOHs are supported by three deputy chief medical officers whom each have responsibility for specific areas, such as health administration and tendering; communicable and non-communicable diseases, reproductive and child health services, and the HMIS [26].

In a district, there are several government departments which provide health services indirectly, in addition to the Health Department itself services. For example, the Department of Women and Child Development has a mandate for nutrition for mothers and children, the Department of Rural Development works towards hygiene and sanitation programmes and the formation of Village Health, Sanitation and Nutrition Committees, and the Department of Education is responsible for the health of adolescents.

**Figure 1:**
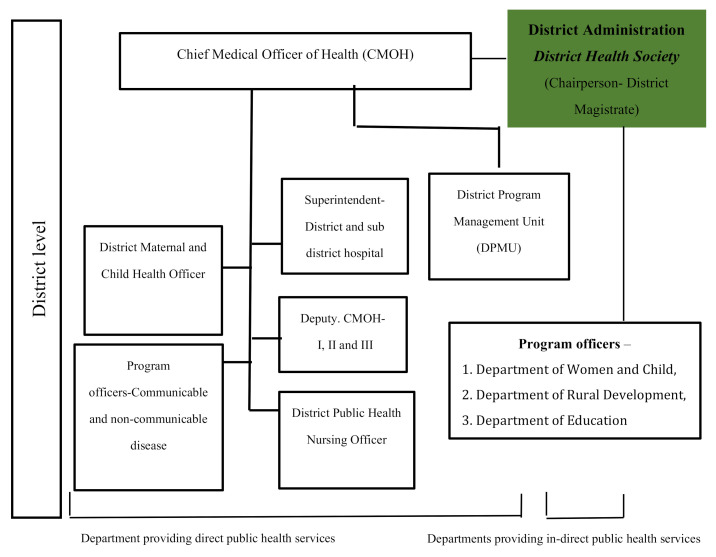
Health system connectivity at the district level in West Bengal.

The District Health Society (DHS) is the primary district-level decision-making forum and facilitates inter-departmental convergence. It is constituted as part of the Health Sector Reform Programme and is the highest policy-making body at the district level, with responsibility for planning and managing all health and family welfare programmes (Figure 1).

The objective of the DHS is “to support the district health administration in an additional managerial and technical capacity for planning and implementing all health and family welfare programmes in the district” (Government of West Bengal, Notification for District health and Family Welfare Samiti, Ministry of Health and Family Welfare, Government of West Bengal; Notification no-HF/SPSRC/112/2013/384, September 2014). The work of the DHS includes reviewing, assisting and supervising all district health programmes, preparing the district health plan and coordinating the activities of the Health Department with activities of all other departments that provide public health services. The District Magistrate is the chairperson of the DHS, which consists of around 25 members, who meet every month (Government of West Bengal, Notification for District Health and Family Welfare Samiti, Ministry of Health and Family Welfare, Government of West Bengal; Notification no-HF/SPSRC/112/2013/384, September 2014). Among the membership are four members from the district administration, 18 members from the Department of Health and one member each from the Departments of Women and Child Development, Rural Development and Education.

A typical DHS meeting agenda includes public health issues which are handled by the Health Department, as well, they conduct joint activities with other departments, like the Departments of Women and Child Development, Rural Development and Education.

In the Indian context, with the official endorsement of autonomous district planning processes involving multiple departments working towards a common goal, and data available for evidence-based decision-making, critical appraisal of how local decision-making happens is important. This qualitative study assessed the health decision-making process at the district level in West Bengal, the extent to which local data were used for decision-making, planning and resource allocation related to MCH services. This paper adds to the existing literature of gaps and challenges in decision-making processes in LMIC settings, particularly the extent of local data-use for resource allocation for maternal and child health.

## 2. Methods

Qualitative data were collected in two districts in the State of West Bengal between June and October 2015: the qualitative approach enabled us to make insights into the decision-making process and the extent of data use decision-making. The data collection methods included observations of decision-making meetings, in-depth interviews with key informants and content analysis of templates from the study districts that are used to collect data for the HMIS (Table 1). We developed an observation checklist to understand how the DHS meetings were conducted; how interaction happened between departments, in terms of data sharing; and how decisions for planning and resource allocation were made. The interview guide included questions on the health administrative process for decision-making, interactions between different government departments for decision-making and planning, the use of data for decision-making and factors deciding resource allocation in a district. The instruments were translated into Bengali and pre-tested in the study area.

### 2.1. Data Collection

After obtaining permission from the State Health Directorate, we selected the districts for this study in close consultation with the State Secretary of Health and also based on health indicators. South 24 Parganas and North 24 Parganas were identified as representative of the state in terms of health system indicators. South 24 Parganas had a lower district dashboard index of 0.4374 (comprised of 16 MCH indicators), compared with North 24 Parganas where it was 0.4924 [27].

Within each district, respondents were selected for semi-structured in-depth interviews from officials involved in the planning process and members of DHS, in consultation with the heads of the health administration and the Chief Medical Officer of Health. The sample included representatives of the health administration and members of the DHS from the public departments, including Health, Women and Child Development, Rural Development and Education, and the district administration, including the District Magistrate. The in-depth interviews were conducted until no significant new responses emerged. The final sample included 24 respondents (Table 1). The interview guide included information on the functioning of the DHS, records of proceedings of DHS meetings, the composition of the DHS, the role and scope of various departments for convergence and improved decision-making, and the maintenance, flow and utilisation of data elements in the district health information system. Open-ended questions were used to capture the information about these aspects. The instrument, developed in English, was translated in Bengali, the local language of the study area. It was pre-tested before finalisation. Trained researchers proficient in English and Bengali, and having knowledge of the local health structure, conducted in-depth interviews. Most of the interviews were conducted in English, and few in Bengali, which were transcribed and translated to English.

Decision-making meetings of the DHS were observed to gain insights into the planning process, the interactions between stakeholders for decision-making and the use of data for planning and resource allocation. Two DHS meeting were observed in each study district. Beyond providing an initial introduction, the research team remained unobtrusive in these meetings.

Given the primary interest to capture the experience of administrative decision-making at the district level, we used a general phenomenological perspective in the development of the semi-structured data-collection guides and analysis [28]. The research team visited different health system levels in the districts to meet the data providers, data managers and programme officers and collected templates of all HMIS and other programme data forms they maintained, both paper-based and electronic to understand the level of compilation.

### 2.2. Data Analysis

We used triangulation of in-depth interviews, observations and content analysis to highlight how local decision-making for health happens at the district level. This triangulation of the three qualitative data collection method allowed us to cross-check and verify findings from each method. The first level of triangulation focused on the existing process, for which data from the in-depth interviews were cross-checked with observation notes from decision-making meetings. Further triangulation was undertaken to cross-check the observation notes from decision-making meetings with the content analysis of the data templates, to show the availability of data in HMIS and whether they were used in decision making.

For all but three of the interviews, respondents gave consent for audio recording. The recordings were transcribed verbatim, and those in Bengali were translated into English. Data were analysed manually by the first author, in collaboration with the other authors, by going through the meeting observation notes and transcripts. Initial *a priori* codes were identified, to which were added emerging themes from the transcripts. The final analysis included two themes and sub-themes. Theme one highlighted the decision-making structure of health districts and the sub-themes focused on (1) the planning and resource allocation process; and (2) interactions between the health administration and other departments for decision-making. Theme two highlighted the use of data for decision-making. The sub-themes looked at (1) data availability and sharing on maternal, neonatal, and child health between the health and non-health sectors; and (2) the extent of data use for planning in the district decision-making platform.

A database of all the data forms that are maintained at the district level was created using Microsoft Access. Each data form was given a unique number and categorised based on its source, the level the form was maintained, data collected and frequency of reporting. Content analysis of the data type in each form was conducted, according to WHO health system categories of service delivery, contextual factors, medical supplies, workforce, governance and finance, to capture the type of data available for different health system levels, the level of data sharing and the flow. We defined a data element as a recorded event or unit in a data collection form. Methodological details of the content analysis are described elsewhere [23].

Ethical approval for the study was obtained from the London School of Hygiene and Tropical Medicine (reference 6088) and the Health Ministry Screening Committee in India (HMSC/2012/08/HSR). Written permission from the State Secretary of Health was obtained, and the cooperation of the heads of district health administration was sought before data collection. All respondents provided written consent and where they gave permission, the interviews were digitally recorded. The anonymity of identity and confidentiality of information was maintained during analysis.

## 3. Results

The following section describes the decision-making process and use of data for decision-making at a district level.

### 3.1. The Health Decision-Making Process in Districts

*Planning and resource allocation process:* Decentralised planning is one of the pillars of India’s Health Sector Reform Programme that was initiated in 2005, creating a decision making space for district-level staff. Resource allocation is based on a District Health Plan, which each district is expected to prepare annually, but districts in West Bengal develop the plans every three years. District plans are submitted for state approval before being sent on to the national level. In districts, there are regular meetings such as a Public Health meeting on 10th of every month, a Reproductive and Child Health review meetings on 20th of every month, and regular District Task Force (polio) and immunisation meetings to discusses the proposals received from the different health units within the district. The District Programme Management Unit coordinates the planning, troubleshooting and preparation of the District Health Plan and budgeting of various health programmes, combining local needs with the state government’s guidelines and priorities.

Respondents mentioned that often that health plans are structured around the State and Central Government’s core agenda of health programmes. The shrinking of the decision making space is illustrated by an imbalance between local health needs and resource allocation: despite local health needs often being reflected in the plan documentation, the resource allocation depends on priorities within the State health programmes. Planning remains top-down, and priorities change as and when the Government changes.

*“Bottom-up approach should be adopted while making district health plans… Suggestions from the community can be considered and discussed …But we have to adhere to the priorities set by* (the) *Government of India and State Government.”*

(Health Department Representative)

Respondents mentioned that the idea of decentralised planning had been protected to some extent by the DHS, as it manages some flexible untied funds.

*“Sometimes, issues related to funds shortage for implementing programmes can be taken care* (of) *by DHS… District specific useful ideas which need funds can be decided at the DHS.”*

(Health Department Representative)

*Interactions between different departments for decision-making:* The CMOH has interactions with other departments, such as Education, Women and Child Development and Rural Development, during the meetings. However, their contribution to decision-making is limited because programmes are already planned according to the State Government’s health agenda. Respondents from district departments mentioned that although inter-departmental interactions are meant to take place for programme planning and data sharing, there are no discussions regarding financial expenditure. This is due to each department spending funds on their own needs, which have already been approved.

*“Interactions with other departments is only need based … otherwise, there are no such regular interactions other than DHS forum.”*

(Health Department Representative)

The respondents emphasised how the DHS, which is supposed to facilitate inter-departmental convergence and to look at district health needs holistically, has not succeeded in bringing all the departments together to make a comprehensive district health plan.

*“Our department is not getting much importance in DHS meetings. One representative from our end just attends the meeting and is not aware of DHS functions... and the health department is also not taking the initiative to motivate us… Our role is poorly defined.”*

(Other department Representative)

### 3.2. Use of Data for Decision-Making 

*Data availability and sharing:* The content analysis of data available in the study districts showed that there were 94 forms in which health data were being collected, with 6170 data elements. The Health Department maintained 78 data forms, with 3814 data elements, whereas departments providing indirect health services, maintained 16 forms, with 2356 data elements. The Health Department maintains data in both paper-based and electronic formats. Data was collected in paper-based registers at the community level, which was reported to the sub-district (block) level, where data were compiled and then sent to various divisions of the Health Department in the district, such as the immunisation, school health, maternal health, communicable and non-communicable diseases divisions.

In a district, data flowed from level I (community: sub-center) to level II (sub-district: primary and community health centers), from where the compiled forms moved to level III (district: central). The Health Department maintained two online portals to keep track of the indicators needed for health decision-making. Facility performance from sub-center to district hospital was managed through HMIS, and the Maternal and Child Tracking System (MCTS) focused on indicators about reproductive, newborn and child health services.

*“HMIS is a structured format with* (a) *specific set of columns (indicators)... all data coming from different divisions can’t be uploaded on HMIS… MCTS are specifically reproductive and child health-based data portal, rather than for other public health programmes.....”*

(Health Department Representative)

The other departments-maintained data in a paper-based format and data sharing with the Health Department could be seen at the sub-district level, where shared indicators included immunisation status and child growth and malnutrition rates. At the district level, there was no policy or process to share data at regular intervals between departments. Although inter-departmental convergence was the mandate of the DHS, no data was shared for joint planning and decision-making.

*“Data sharing between Health and the Department of Women and Child is a major challenge. There is no concordance between these two departments. At the district level, data sharing should be mandatory at DHS.”*

(other department Representative)

### 3.3. Extent of Data Use for Planning and Decision-Making

Triangulating the findings of the in-depth interviews with observations of the DHS meetings, revealed that there was limited data sharing, presentation or discussion based on health indicators. Even if such a debate happened, it was mostly on service delivery such as institutional childbirth and immunisation rates and was based on available data. Generally, the issues discussed and decisions at DHS meetings related to allocating funds for infrastructure development and the need for health awareness and training programmes, and were not backed by the available data.

*“Yes data is useful for planning (e.g., bed occupancy rate). When (the) Mission Director of NHM* (National Health Mission) *visited this hospital found bed occupancy rate at 130%. Then we send the proposal of increasing beds in maternity ward from 85 to 120 and was discussed in DHS meeting...”*

(Health Department Representative)

Discussions on the construction and renovation of the primary health centers and the requirement for additional beds were based on needs as perceived by the district officials, and not supported by data on infrastructure, despite relevant indicators are available and maintained as part of HMIS. Moreover, there is no further analysis of data on linking infrastructure, human resources and supplies data to health outcome indicators, which is a gap area while making a plan. Similarly, data were not analysed and used to discuss programme monitoring or follow-up of the action plan.

*“We are not monitoring our programmes on the basis of our own data... we are not utilising the data... in fact, we are not benefiting from the large volume of data that we are collecting.“*

(Health Department Representative)

*“Data is very much useful while preparing (the) district health plan. Data supports us every time, but it is also true that due to lack of time and inadequate manpower... it is not utilised. Data is a fascinating tool if we use it properly.”*

(Health Department Representative)

There was no utilisation or sharing of data while taking decisions to allocate funds to programmes that were not specified in the District Health Plan. DHS planning is top-down because it follows State and central Government agendas, and local health demands, evident from the data, were not linked to resource allocation.

*“There is no such link between funding and data; in my personal opinion, funding is particular (predefined state guideline) and never linked with data.”*

(other department Representative)

We also triangulated the direct observation with the content analysis of information systems that were available in a district (Table 2), to look at the feasibility of using data for planning, decision-making and resource allocation. MCH-related issues that were discussed in DHS meetings included service delivery issues, health outcomes and infrastructure and supplies. In the four observed DHS meetings, 21 issues were discussed, and action plans were developed, yet decisions on only nine of these issues were based on data. In contrast, our content analysis showing that data for 20 of the 21 issues were maintained and collected at the district level as part of HMIS and MCTS. In addition, the Department of Women and Child Development also maintains data related to malnutrition, which is available at the district level, but not shared with Department of Health, and thus not used during the decision-making meetings.

## 4. Discussion

This study shows that districts have a decision-making structure, including representatives of all relevant government departments, and resources available to support local plans. However, a well-defined decision-making process is lacking, and there are limited interactions between departments for formal data sharing. Triangulating the observation data with that of the content analysis showed that less than half of 21 issues discussed in the district decision-making meetings were based on data, despite relevant HMIS data being available.

Aligning with local priorities is limited by various factors, including the limited use of health data for making plans and decision, lack of coordination among decision-makers and lack of autonomy [1,29]. Decentralised planning is a cornerstone of the Health Sector Reform Programme in India, and aims to empower local district health administrators to make plans based on local health needs. Studies have shown that within a decentralised structure, administrators can make locally relevant plans which can benefit their communities [2,29,30]. This study adds to a body of literature on decision making space: district-level managers are not able to make an efficient allocation of resources as per local health needs [10]. Still, with some degree of flexibility in terms of the resources available to the district administration, local needs can be addressed. At district level in India, there are many other departments apart from the Health Department that provide public health services and work towards achieving common health outcomes in terms of improvement of service coverage for maternal, neonatal and child health care [22]. To have a holistic decision-making process, the DHS provides space for formal interaction between the multiple players. If this formal interaction could be extended to include data sharing across departments, it would help to align the resources available for community health needs and avoid duplication of effort. Hence, an effective process can contribute to developing a holistic health plan at the district level. In similar settings, district staffs have been reported to have inadequate training and understanding of HMIS, and this is a lack of institutionalisation of HMIS, resulting in a lack of integration into everyday activities [16,22,30].

We found that although data are available across district level departments, they were not analysed and used for decision-making. This wastes resources, if data that has been collected is not utilised. There are further ethical implications, as the study has shown a loss of accountability opportunities, inefficiency in the utilisation of the health system resources and ineffective availability of services, hampering fairness and equity [30,31]. This is similar in other LMICs settings where utilisation of local health data for planning is often sub-optimal [30,31,32]. Studies have shown that timeliness and appropriateness in data availability often result in non-utilisation [31]. Moreover, health-care decision-makers have to deal with a large volume of evidence and may or may not have the capacity to use this evidence in decision-making [33]. HMIS provides opportunities for data-based decision-making, particularly within a decentralised health system where local data can help in setting district health priorities and planning, resource allocation and introducing new services or improving existing service delivery as per the needs of the local population [34,35,36].

This study highlights how the existing decision-making forum at the district level could be strengthened through well-defined structured processes, better coordination between different departments and formal sharing of data to develop a holistic health plan. The study result shows the need for contextualised guidelines and job aids, which can enhance data utility alongside formalising interaction between departments. This system-strengthening initiative could support the local administration in evidence-based decision-making, planning and resource allocation based on local health priorities.

This study has limitations in terms of geographical representation, having been conducted in two districts of one state in India, although care was taken to select typical districts with respect to Indian health systems. The study focused on decision-making for maternal, newborn and child health, which may differ from decision-making for non-communicable and infectious diseases. In addition, interactions for joint planning, resource and data sharing between the Government and the private sector were not captured, because the private sector is not represented in the DHS. Observer bias may also have been a factor when recording the proceedings of the meetings, but care was taken to match observations with the minutes of the meeting to reduce the likelihood of bias. Moreover, during the time frame of the study, only four meetings were conducted in the districts, which limited our sample for observations. Lastly, while conducting the content analysis of data available at the district level, the study did not assess the quality of the data that are maintained as part of the HMIS.

## 5. Conclusions

With a decentralised administration and financial autonomy, and the availability of local MCH data, existing forums at the district level in India have strong potential for playing a central role in evidence-based decision-making for planning and resource allocation. The study further highlights the need for a well-defined decision-making process, which could be achieved through capacity-building using sound guidelines and job aids that can facilitate collaboration and data use among stakeholders. This type of package could provide a platform for district health administrations to undertake situation analysis, engage stakeholders, prioritise, develop action plans and follow-up the action plans based on local data.

## Figures and Tables

**Table 1:** Data collection methods.

Methods	Source of Data	Sample in Two Districts
Observations	District decision-making meetings.	4
In-depth interviews	Respondents from the Health Department	16
	Respondents from government departments that provide indirect public health services	6
	Respondents from the district administration	2
Collection of data templates, which contribute to HMIS	94 forms in which health data are collected from each study district 78 forms from the Health Department in each district 16 forms from departments that provide indirect public health services	

**Table 2:** Use of data for decision-making related to maternal and child health issues in the District Health Society (DHS) *.

Health System Categories	Type of Maternal and Child Health Issues Discussed	Use of Data (Yes/No) *	Availability of Data (Yes/No) #	Availability of Indicators in HMIS # (per Month)
Service delivery	1. Immunisation coverage: sub-district percentage	Y	Y	Number of infants 0–11 months who received: OPV1,2,3; BCG; DPT
	2. Institutional delivery: sub-district and facility-based	Y	Y	Number of facility deliveries (including C-sections); number of women discharged under 48 h after delivery
	3. Deliveries: empanelment of private nursing homes under public private partnership scheme	Y	Y	Number of deliveries
	4. Home births: sub-district	N	Y	Number of home deliveries
	5. C-sections: number performed at facility	N	Y	Number of C-Section deliveries
	6. Use of partograph	N	N	Not available
	7. Information Education Communication (IEC), Behaviour Change Communication (BCC) activities conducted for malaria and dengue fever	N	Y	IEC/BCC activities conducted; available, usable etc.
Health outcome	8. Child malnutrition: proportion of underweight children in the district	Y	Y	A number of children with severe acute malnutrition (SAM). Of children weighed, numbers found to be: moderately underweight/severely underweight
	9. Childhood diseases prevalence: sub-district	N	Y	Number of cases of childhood diseases reported
	10. Malnutrition among pregnant women	N	Y	Pregnant women with anaemia: number having Hb level <11, <7 Deaths of mothers due to anaemia, during pregnancy or delivery
	11. Birth weight of newborn	N	Y	Number of newborns weighed at birth; weighing less than 2.5 kg
	12. Maternal mortality rate	N	Y	Mortality details: name, age, sex, village, causes
	13. Newborn and child death rate	N	Y	Mortality details: name, age, sex, village, causes
Human Resouces	14. Shortage of staff, e.g., at sub-district: Accrediated Social Health Activist (ASHA) Facilitator and data entry operators	Y	Y	Number of staff in post, vacancies etc.
	15. Arranging joint home-visits by ASHA and Anganwadi workers (AWW) to pregnant women near their expected delivery date: sub-district	Y	Y	Number of Village Health and Nutrition day (VHNDs) where Auxiliary Nurse and Midwife (ANM), AWW, ASHA present
	16. Data maintenance skills of frontline workers	N	Y	Number of ASHAs fully trained (5 modules—23 days)
	17. Counselling skills, inter-personal communication skills of Frontline workers	N	Y	Number of trained/skilled staff
Infrastructure and Supplies	18. Construction and renovation of primary health centre, requirement of additional beds	Y	Y	Construction of new primary health centres, staff quarters, new MCH complex, neonatal ward
	19. Operationalising new delivery points	Y	Y	Number of facilities where deliveries take place (delivery points). Number of children referred from health facility/delivery point
	20. Referral transport under the Janani Sishu Suraksha Karaykram (JSSK): Nischay Jan ambulance scheme, including three-wheeler motorised vehicles	Y	Y	Number of sub-districts where referral transport service is available. Ambulance type. Number of sick infants transported by referral transport services
	21. Stock out of medicine	N	Y	Stock position: drugs and medical commodities/consumables. Number of ASHAs having regular supplies for drug kits

* Based on observation; # Based on content analysis; Use of data: district-level decision-makers was referring to the local data while taking a decision.; Availability of data: information collected in a structured form and maintained at the district level. VHNDs: Village Health Nutrition Days; ASHA: Accredited Social Health Activists; MCH: maternal and child health.

## Abbreviations

VHND	Village Health Nutrition Days organised once a month aimed at promoting Reproductive Maternal Newborn Child Health initiatives.
ASHA	Accredited Social Health Activists are community health workers recruited under National Rural Health Mission who work as health promoters in the community.
Anganwadi workers	Anganwadi is the basic unit of the Integrated Child Development Services scheme and covers a population of 1000. The workers are females recruited from the local community and trained in non-formal pre-school education, nutrition and health.
JSSK Nischay Yan	Provision of free referral transport to pregnant women under Janani Shishu Suraksha Karyakram

## References

[1] Talukder N., Rob U., Mahabub-Ul-Anwar U. (2008). Lessons Learned from Health Sector Reform: A Four-Country Comparison. Int. Q. Community Health Educ..

[2] Bossert T.J., Beauvais J.C. (2002). Decentralization of health systems in Ghana, Zambia, Uganda and the Philippines: A comparative analysis of decision space. Health Policy Plan..

[3] Somanji H., Barry S., Dram B. (2017). Health systems strengthening: Improving district health service delivery and community ownership and participation. Afr. Health Monit..

[4] La Vincente S., Aldaba B., Firth S., Kraft A., Jimenez-Soto E., Clark A. (2013). Supporting local planning and budgeting for maternal, neonatal and child health in the Philippines. Health Res. Policy Syst..

[5] Mutemwa R.I. (2005). HMIS and decision-making in Zambia: Re-thinking information solutions for district health management in decentralized health systems. Health Policy Plan..

[6] Maluka S., Kamuzora P., SanSebastian M., Byskov J., Ndawi B., Olsen O.E., Hurtig A.K. (2011). Implementing accountability for reasonableness framework at district level in Tanzania: A realist evaluation. Implement. Sci..

[7] Maluka S., Kamuzora P., Sebastian M.S., Byskov J., Olsen O.E., Shayo E., Ndawi B., Hurtig A.K. (2010). Decentralized health care priority-setting in Tanzania: Evaluating against the accountability for reasonableness framework. Soc. Sci. Med..

[8] Roman T.E., Cleary S., McIntyre D. (2017). Exploring the Functioning of Decision Space: A Review of the Available Health Systems Literature. Int. J. Health Policy Manag..

[9] Liwanag H.J., Wyss K. (2019). Optimising decentralisation for the health sector by exploring the synergy of decision space, capacity and accountability: Insights from the Philippines. Health Res. Policy Syst..

[10] Seshadri S.R., Parab S., Kotte S., Latha N., Subbiah K. (2016). Decentralization and decision space in the health sector: A case study from Karnataka, India. Health Policy Plan..

[11] Barros A.J.D., Victora C.G. (2013). Measuring Coverage in MNCH: Determining and Interpreting Inequalities in Coverage of Maternal, Newborn, and Child Health Interventions. PLoS Med..

[12] Pelletier D.L., Frongillo E.A., Gervais S., Hoey L., Menon P., Ngo T., Stoltzfus R.J., Ahmed A.M.S., Ahmed T. (2011). Nutrition agenda setting, policy formulation and implementation: Lessons from the Mainstreaming Nutrition Initiative. Health Policy Plan..

[13] Panda B., Thakur H.P. (2016). Decentralization and health system performance—A focused review of dimensions, difficulties, and derivatives in India. BMC Health Serv. Res..

[14] Mutale W., Chintu N., Amoroso C., Awoonor-Williams K., Phillips J., Baynes C., Michel C., Taylor A., Sherr K. (2013). Population Health Implementation and Training-Africa Health Initiative Data Collaborative Improving health information systems for decision making across five sub-Saharan African countries: Implementation strategies from the African Health Initiative. BMC Health Serv. Res..

[15] Bhattacharyya K., Murray J. (2000). Community Assessment and Planning for Maternal and Child Health Programs: A Participatory Approach in Ethiopia. Soc. Appl. Anthropol..

[16] Garrib A., Stoops N., McKenzie A., Dlamini L., Govender T., Rohde J., Herbst K. (2008). An evaluation of the District Health Information System in rural South Africa. S. Afr. Med. J..

[17] Gething P.W., Noor A.M., Gikandi P.W., Ogara E.A.A., Hay S.I., Nixon M.S., Snow R.W., Atkinson P.M. (2006). Improving Imperfect Data from Health Management Information Systems in Africa Using Space–Time Geostatistics. PLoS Med..

[18] Mate K.S., Bennett B., Mphatswe W., Barker P., Rollins N. (2009). Challenges for Routine Health System Data Management in a Large Public Programme to Prevent Mother-to-Child HIV Transmission in South Africa. PLoS ONE.

[19] Ronveaux O., Rickert D., Hadler S., Groom H., Lloyd J., Bchir A., Birmingham M. (2005). The immunization data quality audit: Verifying the quality and consistency of immunization monitoring systems. Bull. World Health Organ..

[20] Wordl Health Organization (2017). Everybody’s Business: Strengthening Health Systems to Improve Health Outcomes-WHO’s Framework for Action.

[21] Ministry of Health and Family Welfare (2020). National Rural Health Mission: Meetings People’s Health Needs in Rural Areas-Framework for Implementation 2005–2012.

[22] Prasad A.M., Chakraborty G., Yadav S.S., Bhatia S. (2013). Addressing the social determinants of health through health system strengthening and inter-sectoral convergence: The case of the Indian National Rural Health Mission. Glob. Health Action.

[23] Bhattacharyya S., Berhanu D., Taddesse N., Srivastava A., Wickremasinghe D., Schellenberg J., Iqbal Avan B. (2016). District decision-making for health in low-income settings: A case study of the potential of public and private sector data in India and Ethiopia. Health Policy Plan..

[24] Registrar General of India (2014). Sample Registration System Bulletin 2014.

[25] Office of the Registrar General & Census Commissioner (2020). Special Bulletian on Maternal Mortaliry in India 2010–12.

[26] Department of Health & Family Welfare (2017). Governance.

[27] Department of Health & Family Welfare (2017). Health Management Information System State Dashboard-2015–16.

[28] Creswell J.W. (2007). Qualitative Inquiry and Research Design: Choosing among Five Approaches.

[29] Khaleghian P. (2004). Decentralization and public services: The case of immunization. Soc. Sci. Med..

[30] Nyamtema A.S. (2010). Bridging the gaps in the Health Management Information System in the context of a changing health sector. BMC Med. Inform. Decis. Mak..

[31] Abajebel S., Jira C., Beyene W. (2011). Utilization of health information system at district level in jimma zone oromia regional state, South west ethiopia. Ethiop. J. Health Sci..

[32] Akaco E., Erastus M., Edward K., Scott M. (2015). Data Demand and Use in the Health Sector in Central and Eastern Kenya.

[33] Murthy L., Shepperd S., Clarke M.J., Garner S.E., Lavis J.N., Perrier L., Roberts N.W., Straus S.E. (2012). Interventions to improve the use of systematic reviews in decision-making by health system managers, policy makers and clinicians. Cochrane Database of Syst. Rev..

[34] Gill Z., Bailey P.E. (2010). Bottom up and top down: A comprehensive approach to improve care and strengthen the health system. J. Pak. Med. Assoc..

[35] Qazi M.S., Ali M. (2011). Health Management Information System utilization in Pakistan: Challenges, pitfalls and the way forward. BioSci. Trends.

[36] Chitama D., Baltussen R., Ketting E., Kamazima S., Nswilla A., Mujinja P.G. (2011). From papers to practices: District level priority setting processes and criteria for family planning, maternal, newborn and child health interventions in Tanzania. BMC Women’s Health.

